# Health Brokers: How Can They Help Deal with the Wickedness of Public Health Problems?

**DOI:** 10.1155/2017/1979153

**Published:** 2017-05-25

**Authors:** Celeste E. van Rinsum, Sanne M. P. L. Gerards, Geert M. Rutten, Ien A. M. van de Goor, Stef P. J. Kremers

**Affiliations:** ^1^Department of Health Promotion, NUTRIM School for Nutrition and Translational Research in Metabolism, Maastricht University, P.O. Box 616, 6200 MD Maastricht, Netherlands; ^2^Department Tranzo, Tilburg School of Social and Behavioral Sciences, Tilburg University, P.O. Box 90153, 5000 LE Tilburg, Netherlands

## Abstract

**Background:**

The role of health broker is a relatively new one in public health. Health brokers aim to create support for efforts to optimise health promotion in complex or even “wicked” public health contexts by facilitating intersectoral collaborations and by exchanging knowledge with different stakeholders. The current study aimed to explore the role of health brokers, by examining the motivational, contextual, and behaviour-related factors they have to deal with.

**Methods:**

Fifteen professionals from various backgrounds and from various policy and practice organisations were recruited for a semistructured interview. To structure the interviews, we developed the “Health Broker Wheel” (HBW), a framework we then specified with more details derived from the interviews.

**Results:**

We identified seven primary types of behaviour that health brokers need to engage in: recognizing opportunities, agenda setting, implementing, network formation, intersectoral collaboration, adaptive managing, and leadership. Determinants of health brokers' behaviours were identified and categorised as capability, opportunities, motivation, and local or national contextual factors.

**Conclusion:**

The health brokers' role can be seen as an operational approach and is visualised in the HBW. This framework can assist further research to monitor and evaluate this role, and health promotion practitioners can use it as a tool to implement the health brokers' role and to facilitate intersectoral collaboration.

## 1. Background

Many previous studies have reported on the complexity of emerging worldwide public health problems, such as overweight and obesity [1–5]. The causes of overweight and obesity are complex and there are many underlying interactions between the determinants [6–8]. It has proved to be difficult to address these causes with interventions, due to this complexity and the variety of determinants [9].

Addressing such complex or even “wicked” health problems requires a combination of solutions, involving different sectors, such as business, industry, education, spatial planning, public health care, welfare, sports, housing, civil affairs, agriculture, transportation, public safety, and media [10–17]. An integrated or intersectoral approach is often regarded as the optimal way to prevent complex public health problems, such as obesity and socioeconomic health disparities [11, 18–20].

Previous research has shown the benefits of facilitators, change agents, or “catalysts” of change in connecting stakeholders and subsequently stimulating the integrated approach [21–24]. In Netherlands, the role of “health broker” was introduced a few years ago, and several municipalities have now appointed them [21]. Health brokers are social entrepreneurs [25], who can be characterised as change agents [21]. They aim to create support and establish permanent collaborations and encourage knowledge exchange among politicians, policy-makers, private parties, health promotion practitioners and citizens to improve the health of the community, to reduce the number of disadvantaged persons and to optimise evidence coproduction in the prevention of complex public health problems [11, 21, 26]. Health brokers are assumed to operate as “anchoring points” by connecting community problems to policies and services [21, 27]. For instance, they are expected to support obesity prevention by connecting different parties at the local level, such as various municipal government sectors [28]. As such health brokers can facilitate intersectoral collaboration, combine knowledge from different stakeholders and sectors, and actively incorporate evidence into public health policy and practice.

Harting et al. [21] showed that the complexity of health issues and the local situation often impedes the health brokers' role. To date, however, little is known about the factors influencing health brokerage, such as the motivational, contextual, and competence-related factors they have to deal with. In order to be able to examine such factors, it needs to be clear which are the primary behaviours related to the implementation of the health broker role. The present study aimed to explore the role of the health brokers regarding emerging wicked health problems, by examining these primary behaviours and their determinants. We conducted a qualitative study with semistructured individual interviews with various professionals, based on a broad theoretical framework as outlined below.

## 2. Theoretical Framework

**Figure 1:**
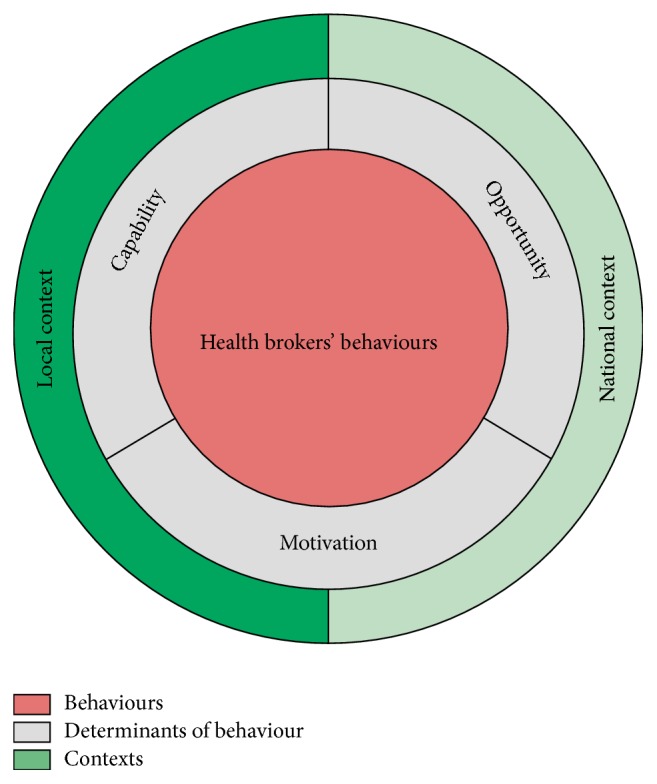
The core of the theoretical framework: the “Health Broker Wheel,” based on Hendriks et al. [29] and Michie et al. [30].

We based our theoretical framework, the “Health Broker Wheel” [HBW] (see Figure 1), on the “Behaviour Change Ball” [BCB] [29], a tool to analyse barriers and facilitators of integrated health policies within local governments [29]. The “Behaviour Change Ball” is in its turn based on the “Behaviour Change Wheel” [30]. In essence, we view behaviours of health brokers as determined by sociopsychological processes that underlie human motivation. This is completely in line with the “COM-B” assumptions in the BCW:* capability*,* opportunity,* and* motivation* (COM) and and* behaviour* (B) [30]. The COM-B system recognizes that behaviour change does not occur in a vacuum but will occur only when COM determinants for health brokerage are sufficiently present [29]. These determinants underlie the implementation of the behaviours [29].

*Capability* refers to what individuals, in this case health brokers, know or are able to do. For example, the ability to guide the process of intersectoral collaboration, to adapt to change and to know about integrated health policies [21, 24, 29–31].* Opportunity* comprises structural variables, including all aspects of the physical and social environment that influence behaviour either directly or through motivation (e.g., through organisational cultures or organisational structures) [29].* Motivation* can involve automatic processes (e.g., beliefs, emotions, and work routines) [32] or more reflective conscious decision-making, such as choices that are made based on evaluations of past experiences [30]. The COM-B system is in turn influenced by different contexts and external influences, including characteristics of both the* national* and* local contexts*.

## 3. Methods

### 3.1. Study Design and Sample

This qualitative study involved semistructured interviews, held between April and June 2013, in which fifteen health promotion professionals from across Netherlands were invited to participate. These professionals had different perspectives on the field of health brokerage. Since the goal of the study was explorative and pioneering, we wanted to interview a wide range of professionals in order to get a relatively broad perspective on relevant behaviours and beliefs, rather than to aim for saturation and deeper information in a small range of interviewees. All professionals were recruited using a snowball method [33]. The inclusion criteria were as follows: having knowledge about the topic of health brokers, based on experiences with or as a health broker, and working in health promotion. The participants were sent an e-mail explaining the topic and goal of the interview. A few days after they had received the e-mail, they were contacted by telephone or e-mail to further explain the study procedure and to set a date for the interview. All of the professionals who were invited agreed to participate in the present study. They were included after they had given permission to record their interview.

The study sample consisted of two health brokers, three former health brokers, two (senior) health policy advisors from the public health services [PHS], one health promotion professional from the PHS, one project leader from the PHS, one former project leader from the National Institute for Health Promotion and Disease Prevention, two PHS managers, two researchers from different universities, and one self-employed public health consultant. This made a total of fifteen interviewees, of which three were men. The health brokers and other professionals were working in a heterogeneous set of municipalities, geographically spread throughout Netherlands. The health brokers were generally structurally embedded in the public health department of their municipality or at the PHS.

### 3.2. Interview Procedure

The interviews were held by the first author at locations chosen by the interviewees. The interview structure (see Appendix) was based on the HBW. Examples of the questions are as follows: “What factors influence a health broker's work?” and “Can you describe the responsibilities of health brokers in your region?” The health brokers were asked additional questions concerning how much they enjoyed their work. The interviews were estimated to take one hour. The core of the HBW (see Figure 1) was developed and applied in this study by starting with the central COM-B system and we filled in the different factors per level (i.e., behaviours, determinants, and contexts) based on the interviews [21, 29].

### 3.3. Data Analysis

The recorded interviews were transcribed verbatim. These transcripts were coded based on the core of the HBW, using NVivo 11.0 software. New thematic codes were made driven by the data. Three interviews were coded by two researchers (CvR and SG). Discrepancies between the two coders were discussed with a third researcher (SK) until agreement had been reached, after which the first author coded the remaining interviews. After the first analyses, the results were summarised for each participant and sent to them for a member check [34]. Only one interviewee made some textual additions.

## 4. Results

### 4.1. Behaviours

**Figure 2:**
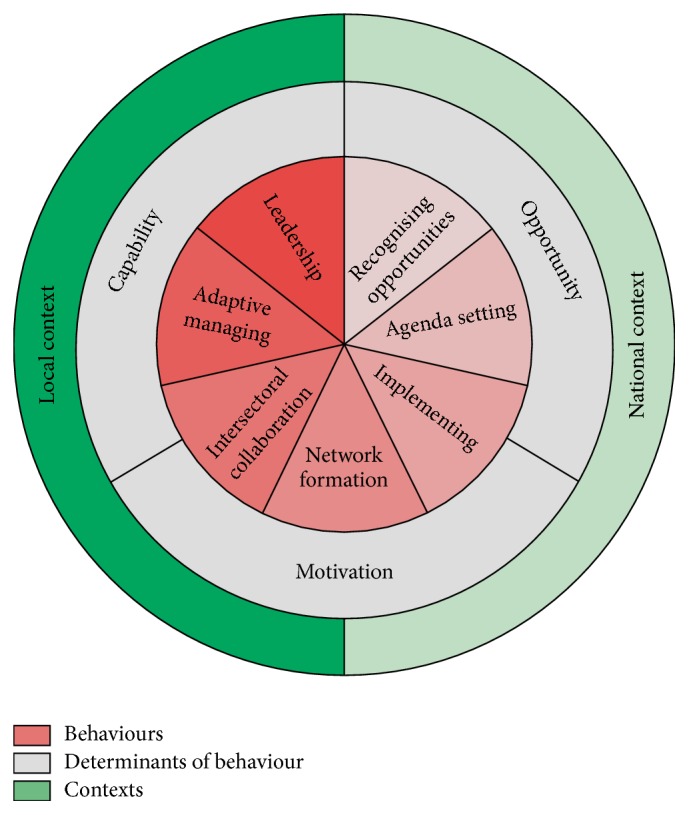
The final version of the theoretical framework: the “Health Broker Wheel.”

Using the interviews data we compiled the inner level (i.e., health brokers' behaviour; see Figure 2). Overall, the interviewees stated that the main task of health brokers is to facilitate* intersectoral collaboration* to improve public health (see Table 1 for an overview of the different health brokers' behaviours and Table 2 for a quotation per each HBW component). They emphasised that intersectoral collaboration is a prerequisite for implementing changes in the physical and social environment, the system, and the policies within a health broker's district. One health broker mentioned that small changes in nonhealth sectors can have a significant impact on health. Topics the health brokers worked on included socioeconomic health disparities, lifestyle themes (e.g., overweight and alcohol consumption), the physical environment (e.g., indoor environment), and loneliness among the elderly.

According to the interviewees, health brokers have to create support and encourage stakeholders to get involved. Participants indicated that many stakeholders do not realise that they can play a part in intersectoral collaboration for promoting health. This is because they do not know exactly how they can play a role in “health,” as that is not their core business. In addition, people work in areas closely related to health and may have different terminologies when talking about health. For example, the Department of Spatial Planning may build more cycle lanes, thereby influencing people's health, but they may not talk about it in terms of health. In order to facilitate* agenda setting* for health in different sectors, the interviewees proposed that health brokers should use more appealing and positive terms, instead of “health” or “prevention.” Furthermore, the health brokers emphasised that the benefits for nonhealth sectors need to be visible for these actors, if they are to participate in intersectoral collaboration.

The interviewees explained that health brokers have a reinforcing task within the network. The core of their binding role is collaborating with practice (e.g., primary care institutions) and policy and with the public health sector. Their job involves identifying the most important health problems* (recognizing opportunities)* and putting them on the political decision-making agenda* (agenda setting)*. Another important task that was mentioned is that of initiating projects or using existing products* (implementing)*. Likewise, health brokers can look for new channels in their district to reach the target group and engage in discussions about health with citizens* (network formation)*. Further important health broker behaviours mentioned by the interviewees were lobbying, completing projects, binding parties, creating support, collaborating, empowering people, adapting to the local context* (adaptive management)*, understanding and speaking the language of the different stakeholders, empathising with others, and focusing on how to make prevention or preventive projects sustainable* (leadership)*, such as advising to make policies. Interviewees also mentioned that if there had been no health brokers, things might have evolved more slowly, because stakeholders would be more likely to stay within their own sector and not collaborate.

### 4.2. Determinants of Behaviour

#### 4.2.1. Capability

Health brokers' competences mentioned by the interviewees include being assertive, being flexible, being patient, not being afraid to be corrected, and knowing how municipal governments work. The respondents stated that it is not sufficient for health brokers to only know about the available health interventions and their scientific foundation but that they should also have knowledge regarding health promotion. However, a background in health promotion was not required, as health brokers do not implement health promotion interventions (at the operational level), but they facilitate these implementations. A background in social science was said to be more useful, because health brokers from outside the health sector can operate more independently. Finally, the complexity of the job, including communication with and switching between (the strategic, tactical, and operational) policy levels, requires highly communicative people with academic skills.

#### 4.2.2. Opportunities

The interviewees reported that successful intersectoral collaboration required resources to be available, such as information, space, time (for stakeholders to collaborate), and funding to implement initiatives. Two respondents claimed that policy plans can make intersectoral collaboration sustainable; nevertheless, the different parties involved must also implement these agreements.

Intersectoral collaboration was said to depend on support from the stakeholders in the community. For example, if one alderman or municipal official leaves the organisation, initiatives can collapse and have to be rebuilt. Furthermore, municipal governments are usually hierarchical, and the internal management is not always well-coordinated. Organisational compartmentalisation (an organisation without internal cross-connections and collaboration) and bureaucracy can hamper intersectoral collaboration.

#### 4.2.3. Motivation

We asked participating health brokers about their motivation towards their work. They all indicated they had a positive attitude towards their role. They liked the collaboration (intersectoral or otherwise), networking, and communication (although communication was also reported to be hard). In their opinion, a health broker should get his/her work enjoyment out of the satisfaction of bringing people together, acquiring new contacts, developing new initiatives, and learning from other people's views.

Negative aspects of their work were, however, also mentioned. Health brokers said that they needed to be patient and sometimes had to let other stakeholders take the lead, which most of them did not like. Another perceived disadvantage was their dependence on other people. Although the health brokers were highly motivated to reduce health problems, their work was not very concrete or visible, which made it hard to keep focusing on the main goal and to enjoy their “successes.” The difficulty of reporting results, such as the fact that results are mostly visible in nonhealth sectors and on the long term, and being accountable for their actions were also mentioned as negative aspects of their work. Furthermore, they mentioned that the work is very wide-ranging and many different stakeholders are involved, which sometimes makes it difficult to retain the overall picture.

### 4.3. Contexts

With the exception of one interviewee, everyone agreed that being a health broker is not so much a job but should be seen as a work attitude, a role, or a working approach. It requires flexibility towards setting goals and taking a demand-driven approach to work. According to the interviewees, a health broker should work and switch between the strategic, tactical, and operational policy levels. Health brokers who switch between the different levels can achieve more than those who work at one of the three levels only. Therefore, the ideal option would be to spread the workload and the three different policy levels over two or three health brokers at all three policy levels in each region.

The interviewees believed the* local context* was an important factor influencing the determinants of health brokers' behaviours. For example, the differences between urban and rural municipalities were considered to be relevant external factors, especially since health problems are often more prevalent and clustered in deprived areas in cities. Interviewees emphasised that in cities there are already many partnerships in place between organisations, which makes intersectoral collaboration easier. On the other hand, there is often more social cohesion in rural areas than in urban areas, which increases the chances of successfully implementing integrated approaches to prevent obesity.

Another aspect of the local context concerns the responsibilities of officials in urban and rural municipal governments. According to the respondents, municipal officials within cities are often responsible only for the public health sector, whereas their counterparts in smaller municipalities have multiple responsibilities in addition to public health. As a consequence, health is typically one of their lowest priorities and less financial resources are available. However, there were also other impediments for health brokers in larger cities. The respondents noted that since urban municipalities have larger numbers of employees, the chances of “serendipity” (beneficial coincidences) are smaller. Therefore, as one interviewee argued, simply having people physically further removed from their colleagues means they will have fewer conversations with them.

The interviewees stated that the governmental cutbacks within the* national context*, which started in 2012, led to national decentralisation, enhanced the responsibilities of municipalities for the health of their citizens [35], and affected municipal budgets, health policy budgets, and intersectoral collaboration. The interviewees believed that the perception of municipal authorities is that prevention of obesity will require a lot of national financial support. However, respondents stated that financial resources can have both a beneficial and an inhibiting impact on the health brokers' work. On the one hand, lack of funding can lead to intersectoral dependence, resulting in cooperation and combining the financial resources. On the other hand, a lack of resources can increase territoriality, which means that people stay within their own sector or area. The lack of resources implies that organisations or sectors (also at the local level) need to be selective in projects or initiatives, which can mean that a health project becomes the last priority. Interviewees mentioned that a “working budget” for health brokers may facilitate the initiation of local activities. This overcomes barriers of territoriality and competent (i.e., highly “capable”) health brokers increase the stakeholders' perceived opportunities to collaborate on the one hand and the motivation to do so on the other.

In addition to financial issues, decentralisation of health care responsibilities, government elections, and the subsequent process of building political coalitions are also influential factors at national level. Furthermore, a former project leader and a manager stated that public-private collaboration is important in integrated efforts to prevent obesity and that this collaboration needs to be strengthened. Public-private collaboration means that private companies, such as health insurers, collaborate with public sector or semipublic sector organisations, such as the PHS. Health brokers can also have a bridge-building or boundary-spanning function in this respect.

## 5. Discussion

The objective of the current qualitative study was to explore the role of health brokers regarding emerging “wicked” public health problems, by examining the primary health brokerage behaviours and the various types of determinants and contexts that influence them. We developed the “Health Broker Wheel” [HBW], which provides a framework for these behaviours and their determinants. Our insights are based on perceptions of closely involved professionals and a survey of the relevant literature. The present study identified that health brokers need to engage in seven different types of behaviour to fulfil their role and that they need to possess certain competences to address wicked health problems. It should be noted that all HBW components interact with each other, both within and between the levels. The levels can rotate relative to each other, which is why the components are presented in the form of a wheel [29, 30].

The inner part of the HBW shows these seven types of health brokers' behaviours.* Recognizing opportunities* means scouting locally for the most urgent health problems, as indicated by both citizens and statistics, and identifying opportunities to tackle a problem.* Agenda setting* is the first stage to prioritize the health problems and to make stakeholders not involved in health care aware of the health problem [29]. Such nonhealth sectors mostly do not realise that they can play a role in promoting health. Hence, health brokers try to get them involved and lobby to put health on the political decision-making agenda. This can be done by reframing the health terminology in such a way that stakeholders understand how they can contribute to the intersectoral collaboration [24, 36]. When health problems are being recognized as important to address, health brokers can* implement* projects to promote health by initiating them, but also by completing them. As an integral part of their work, the health brokers* create networks* by seeking support among stakeholders, encouraging them to get involved, forming a group of various stakeholders and getting these different parties to collaborate across sectors. Health brokers typically guide this process of* intersectoral collaboration*, that is, mediating between and empowering the stakeholders, so that the different parties collaborate and can work as a team [37].* Adaptive managing *means that health brokers adapt their behaviour to the local context and understand and speak the language of the different stakeholders. Important components of this behaviour are empathising with the stakeholders and having an open and learning attitude [29]. Collaborations need to be managed through good* leadership* to formulate a clear vision for addressing health problems [38], to maintain a comprehensive view of all projects and to make sure that prevention or preventive actions will become sustainable in terms of integrated policies.

The most important behaviour expected of health brokers consists of facilitating intersectoral collaboration, for example, between policy and practice in order to improve public health. Previous research has found that intersectoral collaboration and building support are essential behaviours for people in similar positions, such as public managers [21, 39]. In addition to facilitating intersectoral collaboration, the goal of the health brokers' work is to facilitate changes in nonhealth sectors and to make these changes sustainable by means of changes at the policy level. This is comparable to the work of a policy entrepreneur, who links different parties in order to shape policies [40]. Other studies have also identified this strategic, big-picture thinking [39], and long-term perspective [24] as important prerequisites for maintaining intersectoral collaboration [41]. Long-term policies would be most effective in terms of achieving intersectoral collaboration when they are imposed by the national or local government [42]. Long-term policies can also lead to changes in nonhealth sectors, which is beneficial and supportive for the health brokers' role.

The health brokers' job requires multiple* competences* such as being flexible, keeping up with the scientific evidence base in multiple fields, and maintaining contacts with different policy levels and sectors. The competences of health brokers relate to social and interpersonal communication skills, as Koelen et al. [12] and McGuire [39] also stated. Research has demonstrated that “boundary spanners” or similar professionals can use a personal approach to create a shared interest and build social capital and trust [12, 23, 43]. Various studies have shown that trust among stakeholders is essential for teamwork and for building sustainable relationships [12, 23, 27, 39, 44, 45]. Where collaborations already exist, trust is more likely to be building up. However, building trust takes time and occurs throughout the collaboration process [39]. As a consequence, a health broker's main interest in this respect is to initiate collaboration and subsequently ensure its continuation.

Successful intersectoral collaboration is also influenced by* opportunities*. The interviewees indicated that resources and support from stakeholders have a positive effect on intersectoral collaboration and hence on the health brokers' work. One of these resources is time, which is required to build trust and develop policies [12, 24, 46].

Furthermore, health brokers indicated that outcomes of their work are not always visible, which negatively influences their* motivation*, since improvements in health and intersectoral collaboration usually only become visible to other stakeholders in the long term and within nonhealth sectors. Hendriks et al. [36] also addressed problems associated with making outcomes visible in the short term. However, since visibility increases the intrinsic motivation of stakeholders (e.g., funding agencies) to collaborate [12], it is important for health brokers to make short-term successes (on intermediate outcomes) visible and to publicise them.

The national and local contexts were perceived to have a strong influence on the health brokers' work. Factors at the* national level* include decentralisation [35], elections, cutbacks, financial support from the government, policies, health care, developments in society, and public-private partnerships.* Local-level* characteristics that were mentioned included the size of the community, organisational characteristics, social networks, financial resources, public-private partnerships, and current and past collaborations, which impact on the social cohesion in the region (the degree of mutual trust), as was also found in earlier research [39, 41]. As Hendriks et al. [36] stated, municipal managers should be responsible for multiple sectors to facilitate intersectoral collaboration within the organisation. At the same time, Steenbakkers et al. [24] argued that managers should collaborate more with other sectors. The degree to which municipal authorities work in an integrated way may be more important than the differences between urban and rural contexts. Health brokers need to take these factors into account in the different contexts when designing implementation plans for their work.

Collaboration is also influenced by the positioning of health brokers. Health brokers need to switch between the three different policy levels, so it is preferable to have multiple health brokers at all three policy levels within one region, as was also suggested by Harting et al. [21]. Since health brokers' positioning depends on the existing contexts, these contexts must be assessed before positioning health brokers within a region.

### 5.1. Strengths and Limitations

A strength of this study was the use of a theoretical framework to structure the interviews. A limitation was that we did not interview aldermen or local public health policy officers, so the perspective of the municipal authorities is missing. In addition, on average only two professionals were interviewed for each type of job, which does not ensure data saturation. However, since our sample included a variety of professionals working in the policy and practice of Dutch health promotion and who were familiar with the functions of health brokers, we expect that our results provide a well-substantiated view of the health brokers' job. Based on the explorative nature of this study, we opted for interviewing a relatively wide range of professionals to get a broad perspective, rather than to aim for data saturation and more in-depth information from a narrow range of interviewees (e.g., by focusing on health brokers only). Member checks helped improve the reliability of our research.

### 5.2. Recommendations

Countries that aim to engage policy-makers and practitioners in coproduction and coimplementation of health promotion activities may want to include health brokers in their public health system. Since the health brokers' COM-B system has been specified in the HBW, we recommend the use of this framework as a tool when implementing the health brokers' role in a new region. Careful consideration should be given to the question where to position the health brokers and how their positioning fits the complexity of the local context. Furthermore, our study can be seen as a first step in making the health brokers' behaviours more visible. Further studies are needed to provide more in-depth information on (sub)behaviours and their determinants. The HBW could be a useful framework for such studies or for studies addressing other coproduction activities, for example by guiding interview structures in qualitative research.

## 6. Conclusion

Our findings show that health brokers can make useful contributions to address “wicked” public health problems. These problems require intersectoral collaboration and adapting to contextual factors. By operating as “anchoring points” in connecting community problems to policies and services, health brokers represent a good example of bringing together evidence and health policy and practice in addressing complex public health problems. The health brokers' role can be seen as an operational approach and is visualised in the HBW. This framework can help further research to monitor and evaluate this role, and health promotion practitioners can use it as a tool to implement the health broker role and to facilitate intersectoral collaborations.

## Figures and Tables

**Table 1:** Behavioural components of the “Health Broker Wheel.”

Behaviours	Components
Recognising opportunities	(i) Identifying the most important health problems and being aware that citizens can perceive other problems than the statistics indicate (ii) Seeing where opportunities and chances are (iii) Pioneering

Agenda setting	(i) Lobbying (ii) Showing the benefits for nonhealth sectors (iii) Get others to contribute to health (iv) Putting health on the political decision-making agenda

Implementing	(i) Initiating and completing projects

Network formation	(i) Binding parties (ii) Creating support

Intersectoral collaboration	(i) Collaborating (ii) Teamwork (iii) Mediating (iv) Empowering (v) Discussing

Adaptive management	(i) Adjusting to the local context (ii) Speaking the language of different stakeholders (iii) Empathising with others

Leadership	(i) Having a vision for the future (ii) Focusing on sustainable cooperation

**Table 2:** Quotations to illustrate the components of the “Health Broker Wheel”.

Components	Quotations regarding the health brokers' behaviours, their determinants and the different contexts of the health brokers' work
*Behaviours*	

Recognising opportunities	“A health broker in The Hague consistently said: ‘I'm not going to commit myself to a particular activity or theme or whatever. I'm going to look what is going on here, how I can help and how I can convey the residents' wishes to the policy official.'… I thought that this health broker in The Hague had the purest role, because she was not tied to anybody.” (Former project leader)
Agenda setting	“It is important to show that there is a benefit to be gained for the other sector. If you can make that click, then you have somebody on board.” (Former project leader)
Implementing	“We have said to the professionals that if there had been no health broker, things would not have changed, because things would not get started and would not be sustainable.” (Health Broker)
Network formation	“You need to have people who know really intuitively how to get others involved and how to build networks, how to deal with these processes and how to get citizens involved.” (Manager at PHS)
Intersectoral collaboration	“We particularly try to motivate the officials to engage in conversations with other departments and we give them tools to do so.” (Senior policy functionary)
Adaptive management	“I think that you always need to connect with the culture, circumstances and opportunities of a particular setting.” (Health Broker)
Leadership	“I think it is very important that health brokers have the focus on making things sustainable. How do we, if we initiated a couple of things, get people to take autonomy and carry on with it themselves?” (Manager at PHS)

*COM*	

Capability	“Someone who understands what residents' say in their local dialect and who can explain this to the relevant officials.” (Former project leader)
Opportunity	“There has to be willingness on the part of different parties to collaborate. Otherwise, it's like flogging a dead horse. And there has to be willingness to share data.” (Public health consultant)
Motivation	“I liked to initiate the discussion and let people think about ‘What could I do' and ‘What can I contribute to health'.” (Former health broker)

*Context*	

National context	“You can say that major structural barriers are the elections, the fact that health is currently not an issue in the coalition, the fact that the left-wing green party is no longer part of the coalition… Those are stumbling blocks.” (Former health broker)
Local context	“What makes it more difficult is that no two aldermen are the same, and they stay only for four years. If you want to eliminate the socioeconomic health disparities, you need to work in a much longer term. And yes, it is supported by the alderman and the municipal executive. But speeding it up and to keeping it high on the political agendas of all aldermen, that is quite difficult.” (Health broker)

*Note*. COM is an abbreviation of capability, opportunity and motivation.

## Appendix

### Structure of Interviews

*Researchers/Managers*

1. What is your background or relation with health brokers?
2. Can you describe the responsibilities of health brokers in your region?
3. What, according to you, is the added value/goal of the deployment of health brokers, with regard to policy, target group and organisations within the neighbourhood?
4. In your opinion how do health brokers influence health problems and intersectoral care?
5. What factors influence a health broker's work? Can you give both positive and negative examples?
6. How and at what level should health brokers be deployed?
7. How can different contexts be taken into account (urban compared to rural contexts)?
8. What successes have health brokers accomplished? Why are they successes?
9. Which problems arose in the implementation of the health brokers' role? Why did they happen?
10. What would have happened if there had been no health broker?
11. What should be improved in the future with respect to this role?
12. Can you give some advice to a PHS which has only just heard of health brokers; what should they take into account and where should they position health brokers in the organisation?
13. Can you give examples of functions comparable to that of health brokers in other sectors or countries?
14. What would be the ideal picture of a health broker? What would work best?
15. Are there any important other matters you want to mention?
16. Do you know other people I could interview?

*Health Brokers*

1. Can you describe a normal working day? What are your responsibilities?
2. What do you like about your job?
3. What do you not like about your job?
4. What is the most important contribution your work makes?
5. In what way does your work influence the problem of overweight (or other health problems) and intersectoral collaboration?
6. What successes have you, as a health broker, accomplished? Why are they successes?
7. Which problems have you experienced during your work?
8. How can you take into account different contexts (urban compared to rural contexts)?
9. How and at what level should health brokers be deployed?
10. What if there had been no health broker?
11. What should be improved in the future with respect to this role?
12. Can you give some advice to a PHS which has only just heard of health brokers; what should they take into account and where should they position health brokers in the organisation?
13. Can you give examples of functions comparable to that of a health broker in other sectors or countries?
14. What would be the ideal picture of a health broker? What would work best?
15. Are there any other important matters you want to mention?
16. Do you know other people I could interview?

## Additional Points

*Availability of Data and Material*. The transcripts of the interviews are available in Dutch from the corresponding author upon reasonable request.
