# Erratum to “Health Brokers: How Can They Help Deal with the Wickedness of Public Health Problems?”

**DOI:** 10.1155/2018/8784217

**Published:** 2018-02-21

**Authors:** Celeste E. van Rinsum, Sanne M. P. L. Gerards, Geert M. Rutten, Ien A. M. van de Goor, Stef P. J. Kremers

**Affiliations:** ^1^Department of Health Promotion, NUTRIM School for Nutrition and Translational Research in Metabolism, Maastricht University, P.O. Box 616, 6200 MD Maastricht, Netherlands; ^2^Department Tranzo, Tilburg School of Social and Behavioral Sciences, Tilburg University, P.O. Box 90153, 5000 LE Tilburg, Netherlands

In the article titled “Health Brokers: How Can They Help Deal with the Wickedness of Public Health Problems?” [1], reference 35 was incorrectly included as Loketgezondleven.nl. Decentralisatie en lokaal bestuur in Ghana, AGORA Magazine, 2016, 32(1) (doi: 10.21825/agora.v32i1.3654) https://www.loketgezondleven.nl/gezonde-gemeente/gezondheidsbeleid-maken/wettelijke-kaders-gezondheidsbeleid/decentralisatie, while it should be corrected as Loketgezondleven.nl, Decentralisatie overheidstaken (Decentralisation government tasks), 2016, https://www.loketgezondleven.nl/gezonde-gemeente/gezondheidsbeleid-maken/wettelijk-en-beleidskader-publieke-gezondheid/decentralisaties. It has been corrected in place too.
